# Biomarker Signatures of Two Phenotypical Prefrailty Types in the Irish Longitudinal Study on Ageing

**DOI:** 10.3390/geriatrics7020025

**Published:** 2022-02-27

**Authors:** Palina Piankova, Roman Romero-Ortuno, Aisling M. O’Halloran

**Affiliations:** 1 Medical Gerontology, School of Medicine, Trinity College Dublin, D02PN40 Dublin, Ireland; romeroor@tcd.ie (R.R.-O.); aiohallo@tcd.ie (A.M.O.); 2 Mercer’s Institute for Successful Ageing, St. James’s Hospital, D08E191 Dublin, Ireland; 3 Global Brain Health Institute, Trinity College Dublin, D02PN40 Dublin, Ireland

**Keywords:** frailty phenotype, biomarkers, carotenoids, C-reactive protein, lipids

## Abstract

We investigated the biomarker signatures of two previously reported phenotypical prefrailty (PF) types in the first wave of The Irish Longitudinal Study on Ageing (TILDA): PF1 (unexplained weight loss and/or exhaustion) and PF2 (one or two among slowness, weakness, and low physical activity). Binary logistic regression models evaluated the independent associations between available plasma biomarkers and each PF type (compared to robust and compared to each other), while adjusting for age, sex, and education. A total of 5307 participants were included (median age 61 years, 53% women) of which 1473 (28%) were prefrail (469 PF1; 1004 PF2), 171 were frail, and 3663 were robust. The PF2 median age was eight years older than the PF1 median age. Higher levels of lutein and zeaxanthin were independently associated with the lower likelihood of PF1 (OR: 0.77, *p* < 0.001 and OR: 0.81, *p* < 0.001, respectively). Higher cystatin C was associated with PF1 (OR: 1.23, *p* = 0.001). CRP (OR: 1.19, *p* < 0.001), cystatin C (OR: 1.36, *p* < 0.001), and HbA1c (OR: 1.18, *p* < 0.001) were independently associated with PF2, while a higher total (OR: 0.89, *p* = 0.004) and HDL (OR: 0.87, *p* < 0.001) cholesterol seemed to be PF2-protective. While PF1 seemed to be inversely associated with serum carotenoid concentrations and hence has an oxidative signature, PF2 seemed to have pro-inflammatory, hyperglycemic, and hypolipidemic signatures. Both PF types were associated with higher cystatin C (lower kidney function), but no biomarkers significantly distinguished PF1 vs. PF2. Further research should elucidate whether therapies for different PF types may require targeting of different biological pathways.

## 1. Introduction

Frailty is an important concept in geriatrics that is increasingly guiding clinical decision-making and tailored therapeutic interventions for older patients [1]. While several operational definitions of frailty have been proposed, one of the most widely used is the physical frailty phenotype (FP) as defined by Fried et al. [2,3]. This includes a potentially more reversible prefrailty (PF) stage that has attracted much interest due to its greater potential for preventative and health promotion strategies; however, it has been argued that PF may be heterogeneous [4,5].

A previous study based on The Irish Longitudinal Study on Ageing (TILDA) suggested that the PF stage of the FP can be divided into two subtypes with different longitudinal disability trajectories and mortality risks: a lower risk PF1 (unexplained weight loss and/or exhaustion) and a higher risk PF2 (one or two among slowness, weakness, and low physical activity) [6].

To investigate biological differences between the two PF subtypes, we examined available plasma biomarkers in TILDA, many of which are routinely utilized in the diagnosis and/or management of several age-related chronic diseases, which may be associated with the development of frailty. Available biomarkers were C-reactive protein (CRP) as a marker of systemic inflammation [7]; creatinine and cystatin C as markers of chronic kidney disease (CKD) [8]; total, HDL, and LDL cholesterol and triglycerides as markers of cardiovascular disease [9]; and glycated hemoglobin A1c (HbA1c) as a marker of insulin resistance and type II diabetes mellitus (diabetes) [10]. In addition, we examined two dietary carotenoid biomarkers, lutein and zeaxanthin. These are important for macular pigmentation and low levels are linked to the development of age-related macular degeneration (AMD) [11]. These carotenoids have anti-inflammatory and antioxidant properties, and lower levels have also been associated with reduced cognitive function [12]. A deeper understanding of the biological basis of the two PF subdimensions may offer insights into possible early intervention strategies for prefrailty. Therefore, we aimed to investigate the biomarker signatures of the two PF types in TILDA.

## 2. Materials and Methods

### 2.1. Study Sample

TILDA recruited a nationally representative sample of community-dwelling participants aged 50 years and over. The first wave of the study took place between October 2009 and February 2011. The cohort has been described extensively elsewhere [13]. We utilised the TILDA data that is publicly available via the Irish Social Science Data Archive (ISSDA) (http://www.ucd.ie/issda/data/tilda/, last accessed on the 15 July 2021).

### 2.2. Frailty Operationalisation

The FP was operationalised as per Table 1 using TILDA wave 1 population-specific cut-points following the methodology of Fried and colleagues [2]. Weakness was determined by the lowest 20% of sex- and BMI-adjusted grip strength measured on the dominant hand using a baseline dynamometer. Weight and height were measured using standardised procedures during health assessments. Low physical activity was computed as the lowest 20% of sex-adjusted kilocalories (kcals) from the International Physical Activity Questionnaire-Short Form (IPAQ-SF). Slowness (slow walking pace) was measured by the slowest 20% of sex- and height-adjusted time taken in seconds to perform the Timed Up-and-Go (TUG) walking task. Unintended weight loss was ascertained by answering “Yes” to the question, “In the past year have you lost 10 pounds (4.5 kg) or more in weight when you were not trying to?” Exhaustion was captured using two items from the 20-item Centre for Epidemiological Studies Depression (CES-D) scale. Participants were asked how often they felt that “I could not get going” and “I felt that everything I did was an effort”. A response of “moderate amount/all of the time” to either question was considered as being positive for the exhaustion component. The categorical cut points were: 0: robust, 1–2: prefrail, and ≥3: frail.

### 2.3. Prefrailty Operationalisation

**Figure 1:**
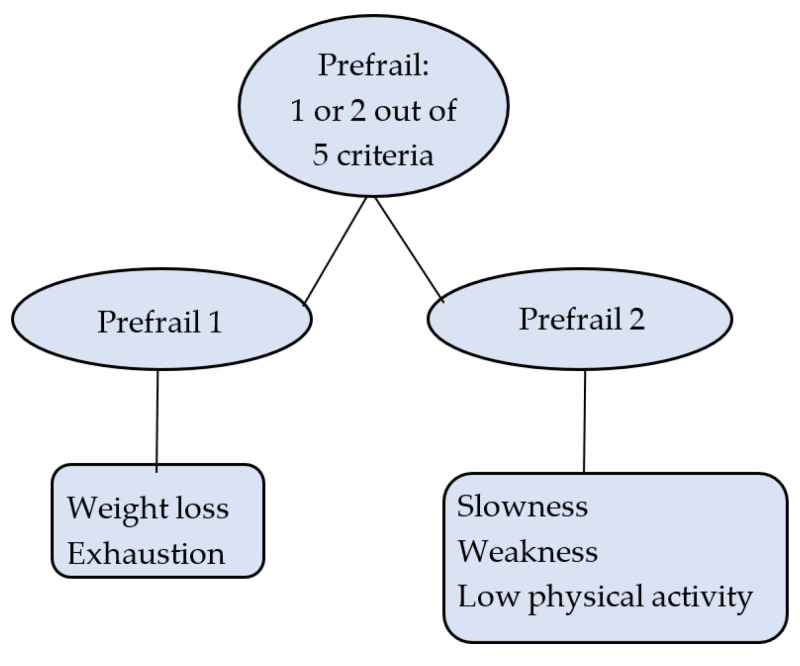
Prefrailty operationalisation.

Prefrailty was separated into two mutually exclusive groups: prefrail 1 (PF1) and prefrail 2 (PF2). PF1 included individuals who were positive for at least one of the following: unexplained weight loss or exhaustion. PF2 included individuals who were positive for one or two of the following: slowness, weakness, and low physical activity (Figure 1).

### 2.4. Biomarkers and Other Measures

During the wave 1 health assessment, trained nurses collected blood samples from participants. The following serum biomarkers were measured: C-reactive protein (CRP), creatinine, cystatin C, HbA1c, total cholesterol, high density lipoprotein (HDL), low density lipoprotein (LDL), triglycerides, zeaxanthin, and lutein. Body mass index (BMI) was measured during the health assessment. Education level was self-reported and coded as follows: any primary schooling (≤8 years) = 0; any secondary school (9–14 years) = 1; university or higher (≥15 years) = 2. Statin use (yes or no) was obtained through questionnaire.

### 2.5. Statistical Methods

All statistical analyses were conducted with Stata 14 MP. Descriptives were given as count with percentage for categorical variables and medians with interquartile ranges (IQR) for continuous variables that were not normally distributed. Bivariate comparisons between PF groups and characteristics of interest were performed with the two-sided Mann–Whitney U test for continuous variables and the chi-square test for categorical variables. Binary logistic regression models evaluated the independent association between biomarkers and PF1 versus robust, PF2 versus robust, and PF2 versus PF1, while adjusting for age, sex, and level of educational attainment. The level of statistical significance was set at *p* < 0.05. Bonferroni multiple test correction across 10 biomarkers for each outcome was set at *p* < 0.005.

We estimated that a sample size of n = 326 in the PF1 group and n = 487 in the PF2 group would detect an effect size of 0.20 with power set at 80% and alpha set at 0.05. Using the Bonferroni-corrected alpha = 0.005 for multiple testing of 10 biomarkers, estimated sample sizes increased to n = 553 in the PF1 group and n = 829 in the PF2 group.

## 3. Results

**Figure 2:**
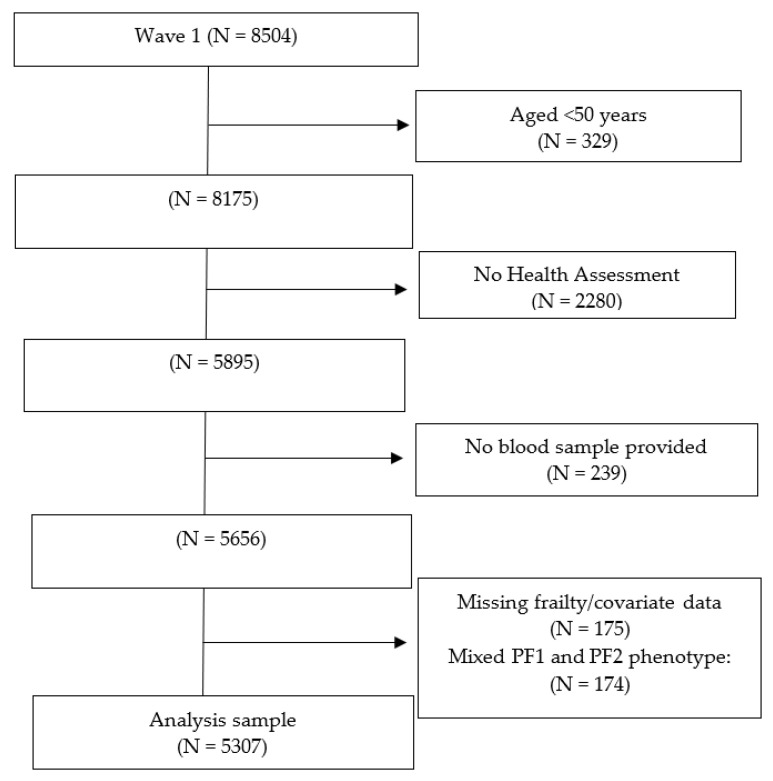
Participants’ flow chart.

Of the total wave 1 sample of 8504 participants, 329 were under 50 years of age, 2280 did not complete the health assessment, and 239 did not provide a blood sample. Of the remaining 5656 participants, 175 had incomplete frailty information and 174 had a mixture of PF1 and PF2 prefrailty components (Figure 2). This left an analysis sample of n = 5307 participants. The median age of the analysis sample was 61.0 (IQR 14.0, range 50–80) years, and 53.4% were women. Of the 1473 (27.8%) prefrail participants, 469 had PF1 and 1004 had PF2.

Table 2 shows participant characteristics across frailty statuses and their respective statistical comparisons with those who were robust, as well as PF1 vs. PF2 comparisons in the last column on the right. Compared to robust, PF1 was associated with lower lutein (*p* < 0.001), zeaxanthin (*p* < 0.01), and creatinine (*p* < 0.05) levels. Compared to robust, PF2 participants were older (*p* < 0.001); less likely to be female (*p* < 0.001); and had higher BMI, CRP (*p* < 0.001), creatinine (*p* < 0.001), cystatin C (*p* < 0.001), and HbA1c (*p* < 0.001); and lower total (*p* < 0.001), HDL (*p* < 0.001), and LDL cholesterol (*p* < 0.001). PF2 also had lower lutein (*p* < 0.001) and zeaxanthin (*p* < 0.001) levels. Compared to PF1, PF2 participants were more likely to be older (*p* < 0.001); less likely to be female (*p* < 0.001); less likely to have third-level education (*p* < 0.001); and have higher BMI (*p* < 0.001) and statin use (*p* < 0.05). They also had higher CRP (*p* < 0.01), creatinine (*p* < 0.001), cystatin C (*p* < 0.001), and HbA1c (*p* < 0.001); and lower total (*p* < 0.001), HDL (*p* < 0.01), and LDL (*p* < 0.001) cholesterol.

As Table 3 shows, compared to robust and after adjusting for age, sex, and education, higher levels of lutein and zeaxanthin were independently associated with a lower likelihood of being classified as PF1 (OR: 0.77, *p* < 0.001 and OR: 0.81, *p* < 0.001, respectively). Higher levels of cystatin C were associated with a higher likelihood of being classified as PF1 (OR: 1.23, *p* = 0.001). On the other hand, higher levels of CRP (OR: 1.19, *p* < 0.001), cystatin C (OR: 1.36, *p* < 0.001), and HbA1c (OR: 1.18, *p* < 0.001) were independently associated with PF2, while higher total (OR: 0.89, *p* = 0.004) and HDL (OR: 0.87, *p* < 0.001) cholesterol seemed to be PF2-protective. No biomarkers significantly distinguished between PF1 and PF2 in the adjusted model.

## 4. Discussion

In this study, we aimed to investigate the biomarker signatures of two PF types in TILDA to gain insights into possible biological differences and early intervention strategies for prefrailty. While PF1 seemed to be inversely associated with serum carotenoid concentrations and hence have an oxidative signature, PF2 seemed to have pro-inflammatory, hyperglycemic, and hypolipidemic signatures. Both PF types were associated with higher cystatin C (lower kidney function), but no biomarkers significantly distinguished PF1 vs. PF2 in the adjusted models. On the other hand, there were demographic differences between PF1 and PF2, with PF2 being older (median 8 years) and less likely to be female.

The PF1 biomarker signature was potentially indicative of low dietary intake and/or absorption of lutein and zeaxanthin, which cannot be synthesized by the body. Reduced levels of these biomarkers would suggest a lower antioxidant and anti-inflammatory reserve. Both lutein and zeaxanthin are plasma carotenoids known to display anti-inflammatory and protective properties against AMD, cognitive decline, and endothelial dysfunction [11,12,14,15,16]. The combination of lutein + zeaxanthin has been reported in several studies as displaying an anti-inflammatory effect but remains to be further studied as it fluctuates, not only with dietary intake/absorption, but with many lifestyle-related and physiological factors that are affected by the ageing processes [16]. Whether these differences are biologically or clinically meaningful is difficult to say and would require prospective or longitudinal analysis.

Regarding renal signatures in both PF types, kidney function has been documented to decline with age as a result of age-related hemodynamic and structural changes [17]. While kidney function (as judged by both cystatin C and creatinine) seemed better in PF1 compared to PF2, the median age for PF1 was 60 compared to 68 for PF2. The association of higher cystatin C with PF2 could be accounted for by this large age difference. The association between PF2 and lower renal function could also be explained given the operational definition of PF2, which includes weakness and slowness that can correspond to the phenotypical manifestation of myopathy secondary to kidney disease [18]. However, in the case of PF1, age was not significantly different compared to the robust, which could hypothetically indicate that PF1 symptoms (exhaustion, unexplained weight loss) may be early symptoms of kidney dysfunction in younger adults.

PF2 seemed to have pro-inflammatory, hyperglycemic, and hypolipidemic signatures. Higher CRP can be associated with chronic inflammatory diseases such as arthritis, which on the previous TILDA main analysis [6] was more common in PF2 participants (37.4% vs. 30.0%, *p* = 0.005). Higher HbA1c being independently predictive of PF2 could also be due to the documented higher proportion of type II diabetes mellitus in the PF2 group in TILDA (11.9% vs. 8.5%, *p* = 0.048) [6].

Regarding the hypolipidemic signatures of PF2, it could also be potentially explained by the 8-year median age difference between the two PF groups. It has been reported that LDL, HDL, and cholesterol all follow an age-related decline [9,19]. In addition, the prescription of statins will affect individual markers of lipid status. In addition to the higher prevalence of diabetes in PF2, PF2 in the previous TILDA report also had a higher prevalence of hypertension (44.5% vs. 39.2%, *p* = 0.049). Indeed, in our analysis, statin prescriptions seemed more frequent in PF2 (38.3% vs. 32.6%, *p* < 0.05, Table 2). Higher mortality has been reported among older people with lower levels of total cholesterol [9,20], which is consistent with the previously reported finding that PF2 had higher mortality in TILDA [6]. Interestingly, higher HDL levels were protective for PF2. HDLs are thought to be atheroprotective and reduce the risk of cardiovascular disease; besides their antioxidant, antithrombotic, anti-inflammatory, and anti-apoptotic properties in the vasculature, HDLs also improve glucose metabolism in skeletal muscle [21].

Strengths of this study include the large sample sizes, the availability of a number of biomarkers that are routinely utilized in clinical practice in the diagnosis and/or management of several age-related chronic diseases (i.e., CRP, creatinine, HbA1c, lipids), and the use of the conservative Bonferroni multiple test correction across biomarkers for each outcome. Limitations of our study include its cross-sectional, observational design precluding the establishment of causality relationships. Using the Bonferroni-corrected alpha = 0.005 for multiple testing of 10 biomarkers, the estimated sample sizes increased to n = 553 in the PF1 group and n = 829 in the PF2 group, suggesting that we may be modestly underpowered for testing in the PF1 group using the Bonferroni method. Even though regression models controlled for age, a large age difference between PF types could still be in a great measure responsible for the differences observed. In addition, different multimorbidity patterns in the two PF groups may also explain their different biomarker signatures. Even though the TILDA ISSDA dataset to which we had access to did not include information on morbidities, we were able to refer our analyses to the main TILDA study conducted on the non-publicly available dataset, which was done on the same cohort [6] and helped better frame our results.

## 5. Conclusions

Cross-sectionally, PF1 and PF2 seemed to have different biomarker signatures compared to robust. Based on our results, we do not have sufficient evidence to recommend the use of carotenoid or renal function biomarkers to screen for the specific identification/prevention of PF1; nor can we recommend systematic screening with CRP, cystatin C, HbA1c, and lipids for the specific identification of physical performance abnormalities related to PF2. Clearly, longitudinal research and/or clinical trials are necessary to explore these and other biomarkers further. In the future, this research may show that prefrailty may be amenable to different therapies aimed at different biological pathways.

## Figures and Tables

**Table 1:** Operationalisation of the frailty phenotype in TILDA.

Fried Criteria	Cut-Offs	Scoring
Weight Loss	“In the past year, have you lost 10 pounds or more?”	Yes = 1
Slowness	Male: Height ≤ 173 cm: gait speed < 109.7 cm/s Height > 173 cm: gait speed < 116.7 cm/s Female: Height ≤ 159 cm: gait speed < 100.7 cm/s Height > 159 cm: gait speed < 108.4 cm/s	Yes = 1
Weakness	Male: BMI < 24: grip strength ≤ 20.5 kg BMI 24–26: grip strength < 21.5 kg BMI > 26: grip strength < 23 kg Female: BMI ≤ 23: grip strength < 11.5 kg BMI > 23: grip strength < 13 kg	Yes = 1
Physical Activity	Male (low on IPAQ-SF per 20th percentile): IPAQ-SF score < 462 Female (low on IPAQ-SF per 20th percentile): IPAQ-SF score < 99	Yes = 1
Exhaustion	“I could not get going” or “Everything I did was an effort”	Yes = 1

BMI: body mass index. IPAQ-SF: International Physical Activity Questionnaire Short Form.

**Table 2:** Associations between frailty groups and characteristics of interest.

	Total N = 5307	Robust ^†^ N = 3663	PF1 N = 469	PF2 N = 1004	Frail N = 171	PF1 vs. PF2 (Absolute Difference)
Age, median (IQR) years	61.0 (14.0)	60.0 (12.0)	60.0 (12.0)	68.0 (17.0) ^c^	73. (18.0) ^c^	8.0 ^c^
Female sex %	53.4	53.4	63.8	48.9 ^c^	52.2 ^b^	14.9 ^c^
Education %						
Primary	25.1	21.3	25.8	34.8	46.8	9.0
Secondary	41.2	42.0	38.6	40.4	36.2	1.8
Third level	33.7	36.7	35.6	24.8	17.0	10.8 ^c^
BMI, median (IQR) kg/m^2^	28.1 (6.00)	27.9 (5.7)	27.9 (6.7)	28.9 (6.4) ^c^	28.3 (8.3)	1.0 ^c^
Statin use %	30.2	27.0	32.6	38.3	43.3	5.7 ^a^
CRP, median (IQR) mg/L	1.67 (2.36)	1.57 (2.09)	1.66 (2.52)	2.00 (3.02) ^c^	3.06 (6.36) ^c^	0.34 ^b^
Lutein, median (IQR) µmol/L	0.181 (0.123)	0.186 (0.124)	0.164 (0.123) ^c^	0.172 (0.125) ^c^	0.130 (0.85) ^b^	0.008
Zeaxanthin, median (IQR) µmol/L	0.045 (0.040)	0.047 (0.041)	0.040 (0.039) ^b^	0.040 (0.038) ^c^	0.031 (0.023) ^a^	0.00
Creatinine, median (IQR) µmol/L	77.0 (23.0)	77.0 (22.0)	74.0 (24.0) ^a^	80.0 (24.0) ^c^	84.0 (40.0) ^c^	6.0 ^c^
Cystatin C, median (IQR) mg/L	0.94 (0.21)	0.93 (0.19)	0.93 (0.20)	1.02 (0.29) ^c^	1.16 (0.50) ^c^	0.09 ^c^
HbA1c, median (IQR) mmol/L	32.0 (5.0)	32.0 (5.0)	32.0 (5.0)	33.0 (6.0) ^c^	34.0 (6.0) ^c^	1.0 ^c^
Total Cholesterol, median (IQR) mmol/L	5.1 (1.4)	5.2 (1.5)	5.2 (1.4)	4.90 (1.4) ^c^	4.6 (1.6) ^c^	0.3 ^c^
HDL, median (IQR) mmol/L	1.48 (0.57)	1.50 (0.57)	1.47 (0.58)	1.41 (0.55) ^c^	1.34 (0.46) ^c^	0.06 ^b^
LDL, median (IQR) mmol/L	2.90 (1.21)	2.92 (1.26)	2.90 (1.40)	2.63 (1.32) ^c^	2.50 (1.30) ^c^	0.27 ^c^
Triglycerides, median (IQR) mmol/L	1.47 (1.11)	1.45 (1.11)	1.53 (1.09)	1.53 (1.14) ^a^	1.49 (0.99)	0.00
Slowness, median (IQR) TUG time, s	8.4 (2.2)	8.2 (1.8)	8.3 (1.9) ^a^	9.9 (4.1) ^c^	14.3 (6.4) ^c^	1.6 ^c^
Weakness, median (IQR) grip strength, kg	24.5 (14.0)	26.5 (14.0)	23.0 (11.5) ^c^	20.5 (15.5) ^c^	16.0 (11.0) ^c^	2.5 ^c^
Physical activity, median (IQR) IPAQ-SF met minutes	1935 (3573)	2586 (3863)	1986 (3366) ^c^	396 (1666) ^c^	0 (198) ^c^	1590 ^c^
Weight loss %	5.6	0.0	46.5	0.00	45.6	46.5 ^c^
Exhaustion %	7.3	0.0	61.2	0.00	58.5	61.2 ^c^

^†^ Robust group was the reference group for statistical comparison with either PF1, PF2, or frail groups; ^a^: *p* < 0.05; ^b^: *p* < 0.01; ^c^: *p* < 0.001. Abbreviations: CRP = C reactive protein; HbA1C = glycosylated hemoglobin; HDL = high density lipoprotein; LDL = low density lipoprotein; TUG = Timed Up and Go; IPAQ-SF = International Physical Activity Questionnaire Short Form.

**Table 3:** Results of the binary logistic regression models adjusted for age, sex, and education.

	PF1 vs. ROBUST			PF2 vs. Robust			PF2 vs. PF1		
	OR	95% CI	P	OR	95% CI	P	OR	95% CI	P
CRP	1.13	1.02–1.25	0.015	1.19	1.10–1.28	<0.001 ^†^	1.04	0.93–1.17	0.471
Lutein	0.77	0.69–0.86	<0.001 ^†^	0.89	0.81–0.96	0.005	1.13	0.99–1.28	0.065
Zeaxanthin	0.81	0.73–0.91	<0.001 ^†^	0.89	0.82–0.97	0.010	1.07	0.94–1.22	0.298
Creatine	1.07	0.94–1.22	0.280	1.06	0.97–1.16	0.174	0.99	0.87–1.14	0.937
Cystatin C	1.23	1.08–1.39	0.001 ^†^	1.36	1.25–1.49	<0.001 ^†^	1.10	0.96–1.27	0.176
Hb1AC	1.09	0.99–1.21	0.095	1.18	1.09–1.27	<0.001 ^†^	1.07	0.95–1.19	0.272
Cholesterol	0.86	0.78–0.96	0.007	0.89	0.82–0.96	0.004 ^†^	1.03	0.92–1.17	0.581
HDL	0.86	0.77–0.96	0.006	0.87	0.80–0.94	<0.001 ^†^	0.99	0.87–1.13	0.915
LDL	0.90	0.81–0.99	0.042	0.91	0.85–0.98	0.015	1.02	0.90–0.15	0.774
Triglycerides	1.08	0.98–1.19	0.104	1.12	1.03–1.20	0.005	1.06	0.94–1.19	0.371

^†^ Significant at *p* < 0.005 following Bonferroni multiple test correction for each outcome. Abbreviations: OR = odd ratio, CI = confidence interval; CRP = C reactive protein; HbA1C = glycated hemoglobin; HDL = high density lipoprotein; LDL = low density lipoprotein.

## Data Availability

We utilised the TILDA data that is publicly available via the Irish Social Science Data Archive (ISSDA) (http://www.ucd.ie/issda/data/tilda/, accessed on the 15 July 2021).
